# First Molecular Survey to Detect *Mycoplasma gallisepticum* and *Mycoplasma synoviae* in Poultry Farms in a Strategic Production District of Sicily (South-Italy)

**DOI:** 10.3390/ani12080962

**Published:** 2022-04-08

**Authors:** Paola Galluzzo, Sergio Migliore, Lucia Galuppo, Lucia Condorelli, Hany A. Hussein, Francesca Licitra, Miriana Coltraro, Sabrina Sallemi, Francesco Antoci, Giuseppe Cascone, Roberto Puleio, Guido Ruggero Loria

**Affiliations:** 1 OIE Reference Laboratory for Contagious Agalactia, Istituto Zooprofilattico Sperimentale della Sicilia, 90129 Palermo, Italy; paola.galluzzo@izssicilia.it (P.G.); luciacond1980@gmail.com (L.C.); hany.ahmed@izssicilia.it (H.A.H.); roberto.puleio@izssicilia.it (R.P.); guidoruggero.loria@izssicilia.it (G.R.L.); 2 Dipartimento Scienze e Tecnologie Biologiche, Chimiche e Farmaceutiche, University of Palermo, 90128 Palermo, Italy; 3 Ragusa Area, Istituto Zooprofilattico Sperimentale della Sicilia, 97100 Ragusa, Italy; francescalicitra15@gmail.com (F.L.); miriana.coltraro@gmail.com (M.C.); francesco.antoci@izssicilia.it (F.A.); giuseppe.cascone60@gmail.com (G.C.); 4 DVM Consultant Poultry Specialists, 97013 Comiso, Italy; medvet80@hotmail.it

**Keywords:** *Mycoplasma gallisepticum*, *Mycoplasma synoviae*, laying hens, duplex real time PCR

## Abstract

**Simple Summary:**

Avian mycoplasmosis is caused by several pathogenic mycoplasmas of which *Mycoplasma gallisepticum* (MG) and *M. synoviae* (MS) are the most important. These bacteria may cause both respiratory disease and synovial infections in poultry, resulting in severe economic losses. The aim of this work was to determine the occurrence of MG and MS among commercial and rural laying hens located in Ragusa province (South Italy), using a duplex real time PCR. Four hundred tracheal swabs were collected from seven commercial and 25 rural farms without any clinical disease history. The prevalence in the studied flocks was 28.6% (commercial) and 40% (rural) for MG, and 42.8% (commercial) and 44% (rural) for MS. The overall prevalence at animal level was 12.5% for MG and 23.25% for MS. Data obtained show a lower prevalence of MG than MS in the studied farms. Moreover, both pathogens were spread in rural and commercial farms underlining the importance of surveillance and control of these infections.

**Abstract:**

Mycoplasmas are recognized as avian pathogens, which may cause both respiratory disease and synovial infections in poultry, resulting in severe economic losses. Our study aims to determine the occurrence of *Mycoplasma gallisepticum* (MG) and *Mycoplasma synoviae* (MS) among commercial and rural laying hens located in Ragusa province (South Italy), using a duplex real time PCR. Four hundred tracheal swabs were collected from seven commercial (200 swabs) and 25 rural (200 swabs) farms without any clinical disease history. Out of 400 swabs collected, 50 (12.5%) and 93 (23.25%) were positive for MG and MS, respectively. In particular, 9 (18%) and 22 (23.65%) positive swabs for MG and MS, respectively, originated from commercial farms, compared to 41 (82%) and 71 (76.34%) obtained from rural farms. Data obtained show a lower prevalence of MG than MS in the studied farms. Moreover, both pathogens were spread in rural and commercial farms. PCR could be concluded as a rapid and sensitive method for the identification of MG and MS in areas where commercial farms that are declared *Mycoplasma-free* and rural flocks coexist. These data highlight the importance of surveillance also in rural poultry to monitoring the occurrence of mycoplasmas strains in strategic productive districts.

## 1. Introduction

Avian mycoplasmosis affects poultry production worldwide; their spread in the farm may cause decrease in the weight gain, low efficiency in feed conversion, loss of egg production, hatchability, increase in embryo mortality, carcass condemnation, additional to veterinary costs that causing enormous economic losses [1,2,3].

Three mycoplasmas species are responsible for infecting aquatic poultry (*M. anatis*, *M. anseris*, and *M. anserisalpingitidis*), while another four (*Mycoplasma gallisepticum*, *Mycoplasma iowae*, *Mycoplasma meleagridis* and *Mycoplasma synoviae*) are known as pathogens of terrestrial poultry. Among this latter group, *Mycoplasma gallisepticum* (MG) and *M. synoviae* (MS) are the most worrying and have the greatest impact on the poultry industry; both pathogens have been included in the listed of notifiable diseases of the World Organization for Animal Health [1].

MG has represented for decades one of the main causes of economic losses for the poultry sector [4]. This issue is still present in some countries of the world such as Egypt and Pakistan [5,6]. In contrast, thanks to the introduction of monitoring plans and routine vaccination, a decrease in MG infection in some European countries has been reported [7].

MS has been traditionally considered a secondary pathogen from a clinical and economic point of view [8]. However, in the last two decades the importance of MS surveillance and control in the commercial poultry industry has increased due the identification of particularly virulent new strains [9].

Respiratory signs including conjunctivitis, coughing and sneezing are the main clinical signs of MG in poultry but the infection can also present in a subclinical form. Characteristic of clinical disease may be the occurrence of nasal exudate, rales and breathing through the partially open beak, unilateral or bilateral sinusitis highlighted by the infraorbital sinuses so swollen as to close eyelids, or the occurrence of conjunctivitis, with foamy ocular exudate [1].

MS infection occurs most frequently as a subclinical upper respiratory infection, but is generally associated with poor growth and significant downgrading of carcasses [9], a decline in egg production [10] and the induction of eggshell apex anomalies [11].

The principal lesion caused by MS is synovitis, which occurs in the synovial tendon sheath and joint synovium [12], causing pale combs, lameness, growth retardation and swelling around the joints in chickens [1].

For both pathogens, the ability to invade the respiratory system is favored by the presence of other respiratory pathogens. Indeed, a synergistic effect has been demonstrated between MG and pathogenic strains of *E. coli* [13]. Furthermore, MS can cause severe airsacculitis when co-infections with other viral or bacterial pathogens occurs [14], but it was also frequently isolated from airsacculitis in broiler flocks MG free [15].

Many studies showed the presence of MG and MS infections in birds, other than chickens and turkeys, including different types of game birds such as pheasants, partridges and quails, wild life birds such as sparrows, house and gold finches, pigeons, crows, flamingos and waterfowl such as ducks and geese [16].

Horizontal and vertical transmission are equally responsible for spreading both MG and MS. The transmission rate in eggs may be influenced both by strain involved and by infection stage (from 3–4 weeks in the acute phase to decrease in the chronic phase) [17]. The transmission of these mycoplasmas can also occur horizontally as a result of direct or indirect contact, by transmission of aerosols or by the introduction of contaminated materials or personnel [16].

Unfortunately, MG and MS may frequently remain in the flock at a subclinical level [18]; for this reason, an accurate and prompt diagnosis is of considerable importance in order to control the spread of the diseases. Although isolation of the pathogen is considered as the gold standard test for the MG and MS diagnosis, this technique is rather difficult as avian mycoplasmas require special growing conditions and a lot of time. Molecular biology tests (PCR, real time PCR) are today the laboratory alternatives to detect and analyze DNA of mycoplasmas with high specificity, sensitivity and rapid response [19]. Real-time PCR has distinct advantages over conventional PCR, such as greater reliability, time needed and prevention of environmental contamination, due to post-amplification analysis. In addition, multiplex PCR compared to single PCR allows for testing a large number of samples in a short of time. DNA identification tests were used for the rapid MG and MS detection and identification from cultures or directly from clinical specimens with a considerable reduction in time [20].

Very little information is available on MG and MS occurrence in Italy [21,22] and none are reported for Sicilian poultry farming; therefore, the present study aimed to determine the molecular occurrence of these two mycoplasmas, using a multiplex real time-PCR in rural and commercial laying-hen farms in the Ragusa province, the most productive district of Sicily (South Italy).

## 2. Materials and Methods

### 2.1. Ethical Statement

The study did not involve any animal experiment. Only sample collection from healthy animals was carried out, consisting of a single swab per chicken. This was needed for the laboratory analyses and did not involve any suffering of the sampled animals. This study was conducted as part of the research project (IZS SI 06/19 RC) approved by the Italian Ministry of Health.

### 2.2. Sampling

The number of laying hens present in Ragusa district according to the Italian National registration database (www.vetinfo.sanita.it, accessed on 3 February 2022) is around 1,483,650. Since no epidemiological data were available, the original experimental design provided for the collection of 385 samples from the overall laying-hens population with an expected prevalence of 50%, according to WinEpi software (http://www.winepi.net/, accessed on 1 March 2022) (5% of precision and 95% confidence level). Nevertheless, given the heterogeneity of the two groups in this study, we applied this criterion only in rural farms considering the worst case scenario. In contrast, to assert the Mycoplasma-free status declared by the companies, we used a different approach in the commercial flocks, namely a freedom survey to check the presence of MG and MS considering an expected prevalence of 2% (95% confidence level). The sample size in both cases was calculated according to EPITOOLS online software, (https://epitools.ausvet.com.au/, accessed on 1 March 2022) [23]. No vaccine prophylaxis was implemented in both kind of flocks.

**Figure 1:**
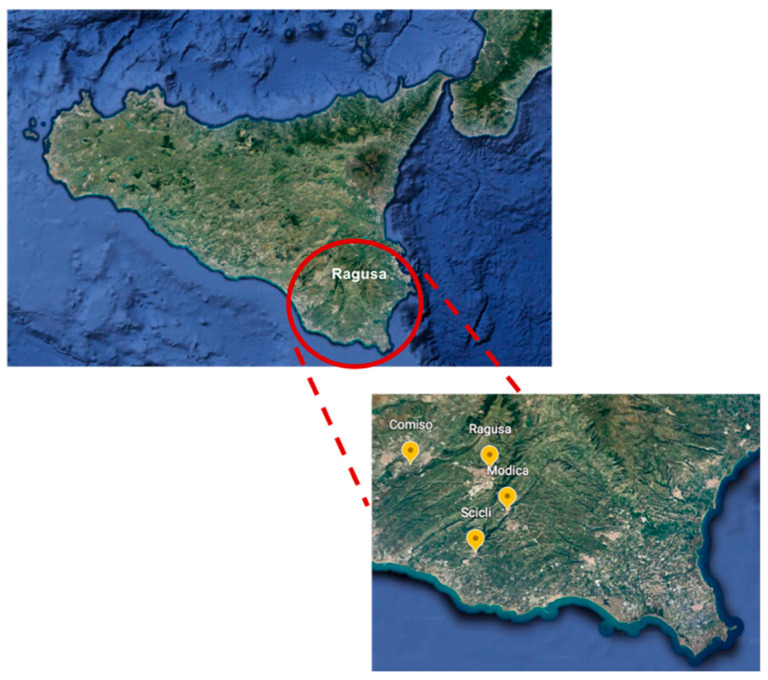
Sample collection area. Samples were collected in four municipalities of Ragusa province (Ragusa, Comiso, Modica and Scicli).

In particular, a total of 400 tracheal swab samples were randomly collected from all 7 commercial (200 swabs) and 25 rural (200 swabs) poultry flocks present in the district. Samples were collected from laying hens without respiratory signs, in the municipality of Ragusa province (Ragusa, Comiso, Modica and Scicli) (Figure 1) during January to December 2021.

The rural flock size ranging from 1 to 60 heads with an age range from 6 months to 3 years old. Hens lived in free-range conditions and were bought from several local retailers. In contrast, commercial flock hens are reared in cages in a shed of 30,000 heads with an age range from 8 to 12 months.

At the sampling time, swabs were placed in tubes with 1 mL of avian Mycoplasma broth medium (Mycoplasma Experience, Redhill, Surrey, UK) and transferred to the Istituto Zooprofilattico Sperimentale della Sicilia in special sterile ice-filled containers to prevent swabs from drying out after sampling.

### 2.3. DNA Extraction and PCR Amplification

Once in the laboratory, swabs were vortexed, and 1 mL of avian Mycoplasma broth medium of each swab was transferred in a 1.5 mL snap-cap Eppendorf tube. All samples were centrifuged for 30 min at 14,000× *g* at 4 °C, the supernatant was carefully removed with a micropipette and the pellet was suspended in 200 μL of PCR-grade PBS (Thermo Fisher Scientific, Rodano, Italy). This suspension was used for DNA extraction using the Quick-DNA Miniprep Plus Kit (Zymo Research, Irvine, CA, USA), following the manufacturer’s instructions suggested for broth cultures. To confirm the presence of MG or MS DNA, a commercial multiplex TaqMan real time PCR was performed. The VetMAX™ Avian *M. gallisepticum* and *M. synoviae* Kit (Thermo Fisher Scientific, Rodano, Italy) was used, following the manufacturer’s instructions. Real-time PCR was performed using the CFX96 Touch Real-Time PCR Detection System (Bio-Rad, Hercules, CA, USA).

## 3. Results

In rural farms, the minimum sample size considering the overall population of 416 heads was calculated in 200 samples. We sampled all heads in the farms with less than 10 units and 50% of heads in the farms with more than 10 units. In contrast, to reach the minimum sample size of 150 units, considering an expected prevalence of 2%, for each commercial farm about 28 hens reared in sheds of 30,000 heads were sampled.

Hens were randomly sampled, and 400 tracheal swabs were collected from 7 commercial (200 swabs) and 25 rural (200 swabs) poultry flocks.

Out of 400 swabs collected, 50 (12.5%) and 93 (23.25%) samples were positives for MG and MS, respectively

**Figure 2:**
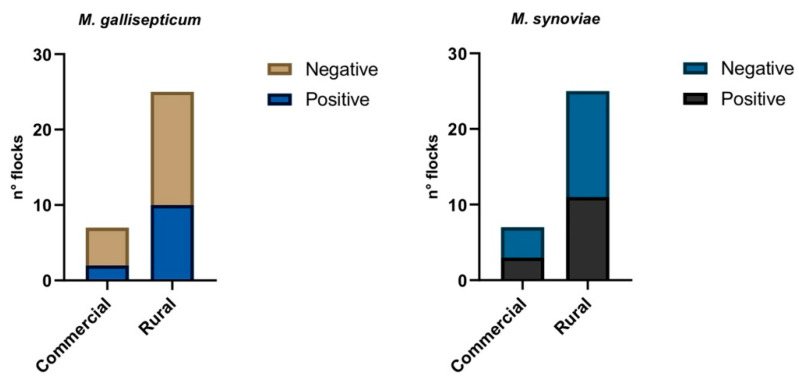
Molecular results obtained by duplex real time PCR to evaluate the presence of MS and MS DNA in sampled flocks.

Moreover, at flock level 2 commercial (28.6%) and 10 rural (40%) flocks were positives to MG, while 3 commercial (42.8%) and 11 rural (44%) flocks were positives to MS (Figure 2).

Table 1 and Table 2 report the prevalence of the two pathogens in sampled rural and commercial flocks, respectively.

At animal level, out of 400 swabs collected, 50 (12.5%) and 93 (23.25%) samples were positives for MG and MS, respectively. In particular, out of the 50 MG positive samples, 41 (82%) and 9 (18%) came from rural and commercial flocks, respectively.

**Figure 3:**
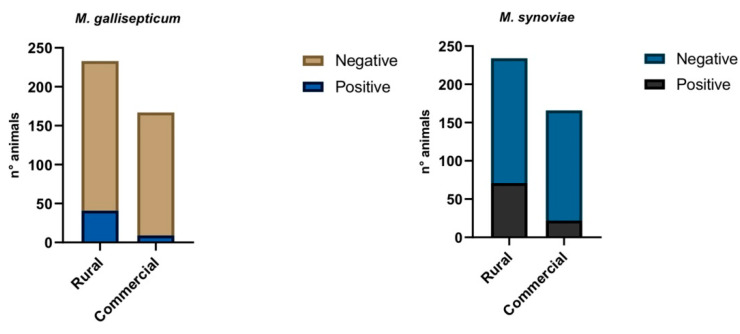
Molecular results obtained by duplex real time PCR to evaluate the presence of MS and MS DNA in swab samples.

Regarding the 93 MS positive swabs, 71 (76.34%) and 22 (23.65%) belonging to rural and commercial flocks, respectively, (Figure 3) were reported.

Finally, co-infection with MG and MS was observed in 23 animals (5.75%) belonging to 7 different rural flocks located in the municipality of Ragusa.

## 4. Discussion

This study provides for the first time preliminary molecular data on MS and MG occurrence in laying-hen farms in Ragusa province, the most productive poultry district of Sicily. MS and MG DNA were detected in rural farms and more surprisingly, in commercial farms declared Mycoplasma-free by the companies, in which we found an unexpected high prevalence ranging from 25 to 37.93 in three different flocks. It is crucial to consider that the sampled hens did not show any clinical signs, which may represent a threat for poultry production since the infection remains silent and can spread without being noticed. It is of great importance that the prevalence in the studied flocks was 28.6% (commercial) and 40% (rural) for MG, and 42.8% (commercial) and 44% (rural) for MS. Although Köhn et al. [24] found, by PCR, a prevalence of 75% of flocks positive for MS and none positive for MG, nevertheless, considering their spread also by vertical transmission [18], and the persistence of these pathogens in the environment [25,26], prevalence rates of MG and MS may increase causing even much higher economic losses to local poultry sector.

The overall prevalence at animal level was 12.5% for MG and 23.25% for MS.

MG prevalence found in this study is lower than other reports: 37.1% in Pakistan [6], 35.5% in Algeria [3] and 56.6% in Kuwait [27]. With regard to the prevalence of MS, our results are in accordance with data reported in the Middle East (Iran) in which 31.2% of tracheal samples collected from hens were found infected with MS in Mazandaran province [28]. Another study carried out in Kerman province reported a MS prevalence of 24.5% in slaughtered ostriches [29].

As reported in the results, MG showed a lower prevalence in the screened farms in contrast to the higher prevalence of MS. The lower prevalence of MG may be due to constant control measures adopted against this well-known pathogenic mycoplasmas.

Conversely, a study conducted in Algeria [3] found a prevalence rates of 35.5% and 18.5%, respectively, for MG and MS in laying hens.

Raising backyard chicken, mainly in the countryside or suburban areas is a common practice in Italy. To date, prevalence of respiratory pathogens as Mycoplasmas in these animals remains unknown. Usually, rural farms lack proper biosecurity measures, their chicken sources are unreliable, and few of them benefit from veterinary care. In the rural farms sampled, there was a high variability in veterinary management and a lack of biosecurity measures. This farming does not concern procedures such as proper disinfection and/or all-in, all-out intervals. In addition, sanitary cleaning and disinfection are often inadequate, causing the persistence of pathogens in the environment. The outdoor breeding also allows contact with wild birds that are often carriers of pathogen mycoplasmas [16].

The presence of MS or MG strains in Italy have already been described in commercial poultry farms [21,22], despite that good biosecurity practices (single-age, all-in all-out farms) should keep these farms free of mycoplasma infection. The potential role of rural poultry farms as reservoir or amplifier of respiratory pathogens and the potential risk for commercial chickens has been already reported [30,31].

The density of the poultry population in the same geographical district where rural and commercial farms coexist represents a not negligible risk factor.

As already described, in Italy [32], we detected a higher presence of MS and MG in rural laying-hen farms, confirming the potential role of this type of breeding to spread mycoplasmas to commercial poultry. The rural and hobbyist farming may not be aware of the biosecurity principles required to keep infectious diseases out and to prevent the spread to other farms.

The poultry sector in the province of Ragusa originated from the significant growth in rural production in the 1950s. The province of Ragusa counts 2,763,047 heads (*Gallus gallus*), equal to 58% of poultry production reared in Sicily (www.vetinfo.sanita.it, accessed on 3 February 2022). In this district together with some modern, industrial farms characterized by high production and good standards of quality and hygiene, there are also several rural poultry farms. Ragusa district plays an important role in Sicilian poultry production, being the biggest producer and the primary exporter of eggs on the island (personal communication). In this context, the sanitary conditions of the poultry flocks are essential for long-term success.

## 5. Conclusions

Our study suggests that the duplex real time PCR used in this study is a rapid and cheap method to detect the occurrence of MG and MS in poultry farming. Nonetheless, this is a useful method for diagnosing these pathogens only in flocks where no vaccine prophylaxis has been performed, as false positives can be obtained.

In addition, this study underlines the importance of surveillance and control of these infections also in rural poultry, in order to prevent the risk of spreading of these subclinical, “hidden” pathogens, which are difficult to eradicate once occurring in the poultry productive chain.

## Figures and Tables

**Table 1:** Prevalence of MG and MG in rural flocks (* average prevalence).

Flock	n° Hens	n° Sample Collected	% MG	% MS
1	10	10	40	80
2	60	30	26.67	30
3	15	8	25	100
4	6	6	0	0
5	30	15	40	33.33
6	5	5	0	0
7	12	6	0	0
8	16	8	12.50	0
9	11	6	33.33	0
10	6	6	0	0
11	42	21	28.57	38.10
12	1	1	0	0
13	10	10	0	20
14	9	9	0	77.78
15	12	6	0	100
16	12	6	100	83.33
17	10	5	0	0
18	20	10	40	80
19	4	4	0	0
20	20	10	0	0
21	10	5	40	40
22	2	2	0	0
23	5	5	0	0
24	3	3	0	33.33
25	3	3	0	66.67
Tot	334	200	20.50 *	35.50 *

**Table 2:** Prevalence of MG and MG in commercial flocks (* average prevalence).

Flock	n° Hens	n° Sample Collected	% MG	% MS
1	30,000	30	0	30
2	30,000	29	0	37.93
3	30,000	28	25	0
4	30,000	27	0	0
5	30,000	30	6.67	0
6	30,000	28	0	7.14
7	30,000	28	0	0
Tot	210,000	200	4.29 *	10.72 *

## Data Availability

Data are contained within the article.
